# Influence of Beef Hot Carcass Weight on Sensory Characteristics of Strip Loin, Eye of Round, and Denver Cut Steaks

**DOI:** 10.3390/foods13060961

**Published:** 2024-03-21

**Authors:** Christina E. Bakker, Samantha R. Egolf, Lydia M. O’Sullivan, Ryan B. Cox, Heather R. Rode-Atkins, Amanda D. Blair, Keith R. Underwood, J. Kyle Grubbs

**Affiliations:** 1 Department of Animal Science, South Dakota State University, Brookings, SD 57007, USA; christina.bakker@sdstate.edu (C.E.B.); lydia.osullivan@sdstate.edu (L.M.O.); amanda.blair@sdstate.edu (A.D.B.); keith.underwood@sdstate.edu (K.R.U.); 2 Department of Animal Science, University of Minnesota, St. Paul, MN 55108, USA; ryancox@umn.edu; 3 Tyson Foods Beef Operations, Springdale, AR 72762, USA

**Keywords:** beef, consumer sensory, Denver cut, eye of round, hot carcass weight, strip steak

## Abstract

The objective of this research was to investigate the influence of beef hot carcass weight (HCW) on consumer sensory attributes. Beef carcasses (n = 116) were selected based on the USDA quality grade and HCW. Lightweight (LW; 296–341 kg), middleweight (MW; 386–432 kg), or heavyweight (HW; 466–524 kg) carcasses with USDA Choice (LC) or USDA Select (SEL) quality grades were used in this study. Carcasses were tracked through fabrication and the semitendinosus, chuck roll, and strip loin were collected and fabricated into eye of round, Denver cut, and strip loin steaks, respectively, for consumer sensory evaluation. USDA Select MW Denver cut steaks had increased overall liking and texture liking scores and were more tender and juicier than the SEL LW steaks (*p* ≤ 0.02). USDA Select MW strip loin steaks had increased overall and flavor liking scores and were more tender than the SEL LW steaks (*p* ≤ 0.02). USDA Choice MW eye of round steaks had increased overall, flavor, and texture liking scores and were juicier than the LW eye of round steaks (*p* ≤ 0.04). The steaks evaluated in this study were differentially impacted by HCW and little to no clear pattern of effects could be determined across cut or quality grade. Additional research is needed to determine the most acceptable HCW from a consumer perspective.

## 1. Introduction

Many factors impact global meat consumption including income, traditions, religious beliefs, and health concerns [1]. Global meat consumption is projected to increase 14% from 2021 to 2030, and population and economic growth are cited as major drivers of this increase [1]. On the surface, these statistics appear to be favorable to the beef industry, however, global beef consumption is predicted to decrease 5% by 2030 [1]. This trend is especially pronounced in countries with high per capita beef consumption as consumers are predicted to shift their protein consumption from beef to poultry [1]. If these predictions hold true and U.S. consumers reduce their frequency of beef consumption, it will be more important than ever for the beef industry to produce beef products with high eating quality to minimize the impact of poor eating experiences on this shift in protein choice.

Meat quality from the consumer’s perspective focuses on the attributes of tenderness, juiciness, and flavor of the cooked product [2]. Tenderness has been shown to be one of the most influential attributes to overall consumer acceptance [3,4]. Because of this, tenderness has been extensively studied [5,6,7,8,9,10]. However, other studies have suggested that flavor is just as important to consumers as tenderness [3,11,12,13]. It is well-established that the palatability characteristics of beef cuts can be influenced by a variety of antemortem and postmortem factors including animal diet [14,15,16,17], the use of growth promoting technologies [18,19,20], and postmortem aging [21,22,23].

The 1991 Beef Quality Audit reported that the average beef hot carcass weight (HCW) was 344 kg [24]. By September 2018, when product collection for this project concluded, the average steer HCW was over 405 kg [25]. According to the 2016 National Beef Quality Audit, 12.4% of carcasses exceeded carcass weights of 453 kg [26]. Furthermore, as cattle have increased in size and carcass weight, chilling methods have not evolved to effectively manage this increase in mass; many packers are still utilizing spray chilling methods adopted in the 1980s and 1990s that were designed for carcasses that were 45 kg lighter [27]. As chilling methods have not changed drastically in the beef industry in recent years, it stands to reason that carcasses with increased carcass weights are at risk of not chilling properly as these systems were designed for smaller carcasses.

Chilling issues have led to altered tenderness and meat quality in beef products [28,29]. Increased protein denaturation and decreased postmortem proteolysis have been observed in deep semimembranosus muscles with slower chilling rates [29]. Moreover, Kim and others [28] investigated heat toughening in strip loins and concluded that increased antemortem temperatures decreased postmortem proteolysis and increased the shear force of loin steaks. However, the impact of carcass weight on beef palatability has been largely unstudied. Therefore, the objective of this study was to evaluate the impact of beef HCW on the consumer acceptability of USDA Choice and Select semitendinosus, serratus ventralis, and longissimus lumborum steaks. We hypothesize that increased hot carcass weight will decrease the consumer acceptability of beef steaks as evaluated by a consumer sensory panel.

## 2. Materials and Methods

### 2.1. Carcass Selection and Sample Processing

Maturity beef carcasses (n = 116) were selected from a commercial packing facility across nine collections between the months of May and September based on USDA quality grade (QG) and HCW. USDA Choice carcasses (LC) with small marbling scores and USDA Select carcasses (SEL) with slight marbling scores were chosen by trained South Dakota State University (SDSU) personnel. Care was taken during selection to not select carcasses with bos indicus (no hump) or dairy influence or yellow fat, indicating grass finishing. Additionally, carcasses were targeted to fit into HCW ranges of 296–341 kg (lightweight [LW]), 386–432 kg (middleweight [MW]) or 466–524 kg (heavyweight [HW]). Based on these grade and weight ranges, carcasses were classified into six groups: LW SEL (n = 20), LW LC (n = 20), MW SEL (n = 20), MW LC (n = 20), HW SEL (n = 16), and HW LC (n = 20). Fewer HW SEL carcasses were chosen due to carcass availability at the packing plant. The carcasses were identified after grading and tracked through fabrication for collection of the *semitendinosus* (Institutional Meat Purchase Specification [IMPS] #171C; n = 114), chuck roll (IMPS #116A; n = 116), and strip loin (IMPS #180; n = 116). Two *semitendinosus* were lost during the fabrication process.

The subprimals were transported under refrigeration back to the SDSU Meat Science Laboratory for further processing. The *semitendinosus* (eye of round) and the *longissimus lumborum* (strip loin) were trimmed to less than 0.6 cm of external fat and fabricated into 2.54 cm steaks. The chuck roll was fabricated to isolate the *serratus ventralis* (Denver cut). The *serratus ventralis* was then bisected along the medial plane and fabricated into 2.54 cm steaks perpendicular to the fiber direction. Three steaks per subprimal were allocated for sensory analysis, vacuum packaged, wet aged at 3 ± 1 °C for 5, 10, or 14-days (aging) postmortem, and frozen at −20 °C until analysis.

### 2.2. Sample Cooking

Steaks were thawed at 3 ± 1 °C for 24 h prior to refrigerated transportation to the University of Minnesota. Steaks were wrapped in aluminum foil and cooked in an electric oven set at 177 °C until an internal temperature of 71 °C was reached, as indicated by a digital thermometer (Cooper-Atkins, Middlefield, CT, USA, Model # DTT361-01). Steaks were trimmed of external fat and connective tissue and cut into 1 cm × 1 cm × 2.54 cm pieces before being placed in porcelain double boilers maintained at approximately 71 °C. Prior to distribution, two pieces of steak per sample code were placed in lidded 118 mL Styrofoam cups and stored in a proofing cabinet (Win-Holt NSF ETL, Syosset, NY, USA, Model #NHPL-1836C) set to 65 ± 5 °C and a humidity setting of 9.

### 2.3. Consumer Sensory Panels

Consumer sensory panels were conducted at the University of Minnesota Sensory Center. Recruiting and experimental procedures were approved by the University of Minnesota Institutional Review Board (IRB STUDY00002408). This study was designed to evaluate the impact of HCW and aging d on consumer acceptability among three beef muscles and two QG, and not to compare between the QG and muscle. Six separate panels were conducted over six days with different panelists, so direct comparisons between panels could not be conducted. Nine sample categories were evaluated in each panel representing the three carcass weight categories and the 3 postmortem aging days. Each sample category was allocated a random 3-digit sample code to blind panelists to the treatments.

The panelist demographics across all six panels are presented in Table 1. Panelists were recruited from students and staff at the University of Minnesota, who were at least 18 years old, had no food allergies or sensitivities, and had consumed cooked beef steak at least yearly.

**Figure 1:**
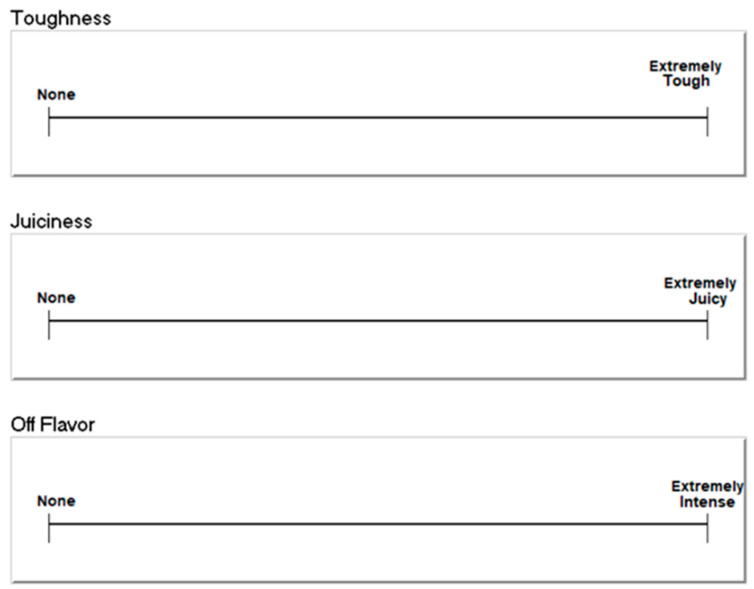
Intensity scales for the evaluation of toughness, juiciness, and off flavor of strip loin, eye of round, or Denver cut steaks.

Samples served to participants were balanced for order and carryover effects. Participants were asked to taste one piece of the sample and rate it for overall liking, flavor liking, and texture liking. Liking ratings were rated on a 120-point labeled affective magnitude scale with the 0 ends labeled “greatest imaginable disliking” and the 120 ends labeled “greatest imaginable liking”. Participants were then instructed to evaluate the second piece for intensity of toughness, juiciness, and off flavor. Intensity ratings were evaluated on a 20-point line scale with the 0 ends labeled “none” and the 20 ends labeled “extremely intense” for off flavor, “extremely tough” for toughness, and “extremely juicy” for juiciness (Figure 1). A score of 0 or “none” indicated that the sample was not juicy, had no off flavor, or was not tough. Sensory data were collected electronically using the SIMS 2000 software program (Sensory Computer Systems, Berkely Heights, NJ, USA).

### 2.4. Statistical Analysis

Data were analyzed using analysis of variance in the GLIMMIX procedure of SAS (v 9.4: SAS Inc., Cary, NC, USA). Sensory traits were evaluated for the effect of aging, HCW, and aging by HCW interactions. Interactions are reported when significant. Sample order was included as a covariate. Participant was considered as a random effect. Treatment least squares means were separated with the PDIFF option of SAS using a significance level of *p* ≤ 0.05. Mean separation tests for all pairwise comparisons were performed using the PDIFF function.

## 3. Results

### 3.1. USDA Choice Denver Steak Sensory

No aging by HCW interactions were observed for overall liking, flavor liking, or off flavor of USDA Choice Denver cut steaks (*p* > 0.05). An interaction was observed for texture liking (*p* = 0.02; Table 2). A HCW by aging interaction was observed (*p* < 0.0001; Table 2) for toughness scores as steaks from the LW and MW carcasses were not impacted by aging, but HW d 14 steaks were more tender than steaks from HW carcasses aged 5 or 10 d. An interaction was also observed for juiciness (*p* < 0.0001; Table 2), with no differences observed throughout aging for the LW or HW carcasses, but steaks from the MW carcasses were found to be juicier when aged 5 or 10 days compared to 14 days.

### 3.2. USDA Select Denver Steak Sensory

No aging by HCW interactions were observed for any of the sensory characteristics measured for USDA Select Denver cut steaks. No differences were detected for flavor liking or off flavor for HCW (*p* ≥ 0.10; Table 3). Hot carcass weight influenced overall liking (*p* = 0.02; Table 3), texture liking (*p* < 0.01; Table 3), toughness (*p* < 0.0001; Table 3), and juiciness (*p* < 0.0001; Table 3). Overall liking and texture liking were increased for steaks from the MW carcasses compared to steaks from the LW carcasses (*p* = 0.04 and *p* < 0.01, respectively). Steaks from the LW carcasses were found to be tougher than steaks from the MW (*p* < 0.0001) or HW carcasses (*p* < 0.01). Heavy weight carcasses (HW vs. MW, *p* = 0.02; HW vs. LW, *p* < 0.0001) produced the juiciest steaks followed by MW then LW carcasses (MW vs. LW, *p* < 0.01).

Aging did not impact (*p* > 0.05; Table 4) the overall liking, flavor liking, juiciness, or off flavor for USDA Select Denver steaks. Texture liking was decreased (*p* < 0.01; Table 4) for steaks aged 5 days compared to 10 or 14 days. As expected, steaks aged for 5 days were tougher (*p* < 0.0001; Table 4) than steaks aged for 10 or 14 days.

### 3.3. USDA Choice Strip Loin Steak Sensory

A main effect was observed for flavor liking (*p* = 0.03; Table 5) of different HCWs with steaks from MW carcasses having increased scores compared to steaks from LW carcasses. No impact of HCW was observed for off flavor (*p* = 0.42; Table 5), and no impact of aging was observed for flavor liking (*p* = 0.75; Table 6) or off flavor (*p* = 0.72; Table 6).

An aging by HCW interaction was observed for overall liking of USDA Choice strip loin steaks (*p* = 0.04; Table 7). Steaks from LW carcasses aged 10 days tended (*p* = 0.08) to have increased liking scores compared to steaks from LW carcasses aged 5 days. An interaction was also observed for texture liking (*p* = 0.01; Table 7). No differences were observed between aging d or weight categories for the HW and MW carcasses. Steaks from LW carcasses aged 10 days had increased texture liking scores compared to steaks aged 5 days. An interaction for toughness (*p* < 0.01; Table 7) was observed. Steaks from LW carcasses aged 5 days were tougher than steaks from LW carcasses aged 10 days and steaks from MW carcasses regardless of aging d. Steaks from HW carcasses at any aging point and LW carcasses aged 14 days were intermediate. An interaction for juiciness was also observed (*p* = 0.01) with steaks from LW carcasses aged 10 days being juicier than steaks from LW carcasses aged 5 or 14 d or from HW carcasses aged 5 days.

### 3.4. USDA Select Strip loin Steak Sensory

A HCW main effect was observed for toughness (*p* < 0.001; Table 5), with steaks from the LW or HW carcasses being tougher than steaks from the MW carcasses. No HCW main effects were observed for overall liking, flavor liking, or off flavor (Table 5).

An aging effect was observed for overall liking (*p* = 0.01; Table 6), flavor liking (*p* = 0.01; Table 6), and toughness (*p* = 0.02; Table 6). Steaks aged 10 days had increased overall liking and flavor liking scores and were more tender than steaks aged 5 days. No impact of aging was observed for off flavor.

A HCW by aging d interaction was observed (*p* = 0.01; Table 7) for the texture liking of USDA Select strip loin steaks. Steaks from HW carcasses aged 10 days tended to have increased texture liking scores than the steaks from LW carcasses aged 14 days (*p* = 0.06) and steaks from HW carcasses aged 5 days (*p* = 0.08). An interaction was also observed for juiciness (*p* = 0.03; Table 7), with steaks from MW carcasses aged 5 days being juicier than steaks from HW carcasses aged 5 days and all other steaks being intermediate.

### 3.5. USDA Choice Eye of Round Steak Sensory

A main effect of HCW was observed for overall liking (*p* < 0.01; Table 8), flavor liking (*p* = 0.04; Table 8), texture liking (*p* < 0.001; Table 8), and juiciness (*p* < 0.0001; Table 8). Steaks from the MW carcasses had increased overall liking, flavor liking, and texture liking scores compared to the LW carcasses. Additionally, steaks from the MW and HW carcasses were juicier than steaks from the LW carcasses. No effect of HCW was observed for off flavor (*p* = 0.51; Table 8). No impact of aging was observed for overall liking, flavor liking, texture liking, juiciness, or off flavor (*p* > 0.23; Table 9)

A HCW by aging interaction was observed for toughness (*p* < 0.05; Table 10), with steaks from LW carcasses aged 5 days being tougher than steaks from MW carcasses aged 5 days and HW carcasses aged 5 or 10 days.

### 3.6. USDA Select Eye of Round Steak Sensory

No impact of HCW was observed for overall liking, flavor liking, or texture liking (*p* ≥ 0.10; Table 8). A HCW effect was observed for off flavor (*p* < 0.01; Table 8), with steaks from LW carcasses having a more intense off flavor compared to HW carcasses.

Aging impacted overall liking, flavor liking, and texture liking (*p* = 0.02; Table 9). Steaks aged 14 days had increased liking scores compared to steaks aged 10 days. No impact of aging was detected for off flavor (*p* = 0.70; Table 9).

A HCW by aging interaction was observed for toughness (*p* < 0.01; Table 10). Steaks from MW carcasses aged 14 days or HW carcasses aged 5 days were less tough than steaks from HW carcasses aged 10 days. Additionally, a HCW by aging interaction was observed for juiciness (*p* = 0.02; Table 10), with steaks from MW carcasses aged 14 days being juicier than steaks from any HCW aged 5 or 10 days. Steaks from HW carcasses aged 10 days were less juicy than steaks from any weight category aged 5 days.

## 4. Discussion

Limited peer-reviewed literature is available that has directly evaluated the impact of hot carcass weight on the sensory attributes of beef. Additionally, minimal research has been conducted to evaluate consumer sensory preferences for the Denver cut. The influence of HCW on tenderness has not been clearly defined. Sañudo and others [30] reported that cattle slaughtered at a live weight of 530–560 kg produced more tender steaks than light weight cattle slaughtered at 300–350 kg. A study conducted on Bonsmara cross steers evaluated the influence of carcass weight on the meat quality attributes of color, drip loss, proteolytic enzyme activity, sarcomere length, myofibrillar fragment length, and shear force on a section of the longissimus lumborum [31]. The authors observed a decrease in shear force values for heavyweight carcasses at 3 d postmortem, but those differences were negated by day 14. Additionally, Lancaster et al. [32] observed no differences in Warner-Bratzler shear force values of semimembranosus steaks from average (340–409 kg) or oversized (≥454 kg) carcasses.

Glanc et al. [33] evaluated the impact of production system and slaughter weight on the beef quality of the longissimus and semitendinosus muscles. They observed no differences in shear force or cook loss between steers slaughtered at a live weight of 545 or 636 kg. However, this study also had an at-home consumer evaluation component of longissimus steaks aged 14 days. The consumers found steaks from the heavyweight group to be more tender, juicier, more beef-like in flavor, and had an increased overall acceptability score. It is worth noting that the steers in the study had HCW of 319.4 and 371.4 ± 1.49 kg for the lightweight and heavyweight categories, respectively. Thus, the lightweight carcasses would fall into the current study’s LW category, but the heavyweight carcasses would fall between the LW and MW categories. In the current study, a HCW by aging interaction was detected for USDA Choice strip loin steaks with steaks from LW carcasses aged 5 days being rated tougher than the LW steaks at 10 and 14 days and all MW carcasses. All weight categories of the USDA Choice strip loin steaks had similar toughness ratings by 14 days of aging.

Beef flavor is complex and is impacted by a variety of factors both pre-harvest and post-harvest [34]. Animal diet, particularly grass-finishing versus grain-finishing, has been shown to impact the fatty acid composition of beef, thus altering its flavor [35,36,37]. Additional factors of animal age, growth technologies, and days on feed may also impact beef flavor [34]. However, the carcasses selected for this study were chosen at the hot carcass scale, so no live animal management traits were known. However, care was taken during selection to not select carcasses with bos indicus influence (no hump) or yellow fat, and only ‘A’ maturity carcasses were chosen at grading.

One of the most influential factors on consumer sensory attributes is quality grade [36,38,39]. An evaluation of consumer sensory scores for four beef muscles—gluteus medius, longissimus lumborum, semimembranosus, and serratus ventralis—in two quality grades, Upper 2/3 (Top) Choice and Select, found that the tenderness, flavor, and overall liking scores were increased for Top Choice steaks compared to Select regardless of the muscle evaluated [39]. However, due to the nature of the panels conducted in the current study, comparisons between quality grades were not possible.

A common theme among the data collected in this study is that when differences in sensory attributes were detected between weight categories, the LW carcasses were generally less desirable than the MW carcasses, and the HW carcasses were intermediate. Unsurprisingly, when an influence of aging was detected, steaks aged 5 days were generally less desirable than steaks aged 10 or 14 days. The positive influence of increasing aging days, up to day 28, on sensory acceptability is well-defined [40,41,42].

## 5. Conclusions

The muscles evaluated in this study were differentially impacted by HCW and aging. No clear pattern of effects could be determined across cut or quality grade. As little to no peer-reviewed research has been conducted to evaluate these specific attributes, these data will serve as a foundation for future research. Additional research must be conducted to determine the mechanisms by which the sensory attributes are impacted in these cuts and potentially establish the most acceptable carcass size or carcass management practices to maximize consumer acceptability.

## Figures and Tables

**Table 1:** Demographic data for consumers evaluating strip loin, eye of round, or Denver cut steaks aged 5, 10, or 14 days.

Panel	Weekly ^1^	Monthly ^1^	Yearly ^1^	Female	Male	Total
**Denver cut**						
USDA Select	58 (53.2%)	50 (45.9%)	1 (0.9%)	78 (71.6%)	31 (28.4%)	109
USDA Choice	61 (58.7%)	41 (39.4%)	2 (1.9%)	77 (74.0%)	27 (26.0%)	104
**Strip loin**						
USDA Select	55 (48.2%)	57 (50%)	2 (1.8%)	85 (74.6%)	29 (25.4%)	114
USDA Choice	54 (51.9%)	47 (45.2%)	3 (2.9%)	78 (75.0%)	26 (25%)	104
**Eye of Round**						
USDA Select	45 (40.5%)	61 (55.0%)	5 (4.5%)	80 (72.1%)	31 (27.9%)	111
USDA Choice	45 (43.3%)	57 (54.8%)	2 (1.9%)	76 (73.1%)	28 (26.9%)	104

^1^ Frequency of beef consumption.

**Table 2:** The interaction of aging and HCW on the sensory attributes of USDA Choice Denver cut steaks as rated by consumers.

Attribute	LW 5 ^1^	LW 10 ^1^	LW 14 ^1^	MW 5 ^1^	MW 10 ^1^	MW 14 ^1^	HW 5 ^1^	HW 10 ^1^	HW 14 ^1^	SEM ^2^	*p*-Value
Texture Liking ^3^	69.8	73.4	73.8	74.3	73.4	70.7	69.1	68.8	75.3	2.11	0.02
Toughness ^4^	9.7 ^a^	8.3 ^ab^	8.1 ^ab^	8.4 ^ab^	8.3 ^ab^	9.3 ^a^	9.1 ^a^	9.4 ^a^	7.0 ^b^	0.54	<0.0001
Juiciness ^4^	8.7 ^abc^	10.3 ^a^	8.8 ^abc^	9.9 ^ab^	10.0 ^ab^	8.1^c^	10.1 ^ab^	8.5 ^bc^	9.8 ^ab^	0.53	<0.0001

^1^ Wet aging days postmortem; LW = Lightweight (296–341 kg), MW = Middleweight (386–432 kg), HW = Heavyweight (466–524 kg). ^2^ Largest SEM reported of least squares means. ^3^ 0 = Greatest imaginable disliking; 120 = Greatest imaginable liking. ^4^ 0 = None; 20 = Extremely tough, extremely juicy. ^abc^ Within a row, least squares means without a common superscript differ (*p* < 0.05).

**Table 3:** The effects of hot carcass weight on the sensory attributes of USDA Choice and USDA Select Denver cut steaks as rated by consumers.

Attribute	LW ^1^	MW ^1^	HW ^1^	SEM ^2^	*p*-Value
**USDA Choice**					
Overall Liking ^3^	74.0	74.3	73.5	1.32	0.84
Flavor Liking ^3^	74.3	75.4	73.6	1.30	0.37
Off Flavor ^3^	4.8	4.8	4.4	0.23	0.12
**USDA Select**					
Overall Liking ^3^	70.2 ^b^	73.4 ^a^	73.2 ^a^	1.30	0.02
Flavor Liking ^3^	71.1	72.4	73.9	1.29	0.10
Texture Liking ^3^	67.8 ^b^	72.6 ^a^	70.5 ^ab^	1.49	<0.01
Toughness ^4^	9.8 ^a^	8.5 ^b^	8.8 ^b^	0.31	<0.0001
Juiciness ^4^	8.4 ^c^	9.5 ^b^	10.3 ^a^	0.31	<0.0001
Off Flavor ^4^	4.8	5.0	4.9	0.25	0.88

^1^ LW = Lightweight (296–341 kg), MW = Middleweight (386–432 kg), HW = Heavyweight (466–524 kg). ^2^ Largest SEM reported of least squares means. ^3^ 0 = Greatest imaginable disliking; 120 = Greatest imaginable liking. ^4^ 0 = None; 20 = Extremely intense off flavor, extremely tough, extremely juicy. ^abc^ Within a row, least squares means without a common superscript differ (*p* < 0.05).

**Table 4:** The effects of aging on the sensory attributes of USDA Choice and USDA Select Denver cut steaks as rated by consumers.

Attribute	5 ^1^	10 ^1^	14 ^1^	SEM ^2^	*p*-Value
**USDA Choice**					
Overall Liking ^3^	73.3	73.7	74.7	1.32	0.54
Flavor Liking ^3^	74.7	74.2	74.4	1.30	0.92
Off Flavor ^4^	4.7	4.8	4.5	0.23	0.54
**USDA Select**					
Overall Liking ^3^	71.0	73.1	72.6	1.31	0.22
Flavor Liking ^3^	72.8	73.6	71.0	1.29	0.12
Texture Liking ^3^	67.6 ^b^	71.4 ^a^	71.8 ^a^	1.49	<0.01
Toughness ^4^	10.2 ^a^	8.8 ^b^	8.1 ^b^	0.31	<0.0001
Juiciness ^4^	9.2	9.2	9.8	0.31	0.08
Off Flavor ^4^	4.8	4.8	5.1	0.25	0.28

^1^ Wet aging days postmortem. ^2^ Largest SEM reported of least squares means. ^3^ 0 = Greatest imaginable disliking; 120 = Greatest imaginable liking. ^4^ 0 = None; 20 = Extremely intense off flavor, extremely tough, extremely juicy. ^ab^ Within a row, least squares means without a common superscript differ (*p* < 0.05).

**Table 5:** The effects of hot carcass weight on the sensory attributes of USDA Choice and USDA Select strip loin steaks as rated by consumers.

Attribute	LW ^1^	MW ^1^	HW ^1^	SEM ^2^	*p*-Value
**USDA Choice**					
Flavor Liking ^3^	70.3 ^b^	73.6 ^a^	71.8 ^ab^	1.23	0.03
Off Flavor ^4^	4.1	3.8	4.0	0.25	0.42
**USDA Select**					
Overall Liking ^3^	69.5	71.4	70.5	1.23	0.28
Flavor Liking ^3^	70.7	71.5	71.5	1.15	0.74
Toughness ^4^	9.2 ^a^	7.9 ^b^	8.7 ^a^	0.31	<0.001
Off Flavor ^4^	4.6	4.3	4.3	0.23	0.43

^1^ LW = Lightweight (296–341 kg), MW = Middleweight (386–432 kg), HW = Heavyweight (466–524 kg). ^2^ Largest SEM reported of least squares means. ^3^ 0 = Greatest imaginable disliking; 120 = Greatest imaginable liking. ^4^ 0 = None; 20 = Extremely intense off flavor, extremely tough. ^ab^ Within a row, least squares means without a common superscript differ (*p* < 0.05).

**Table 6:** The effects of aging on the sensory attributes of USDA Choice and USDA Select strip loin steaks as rated by consumers.

Attribute	5 ^1^	10 ^1^	14 ^1^	SEM ^2^	*p*-Value
**USDA Choice**					
Flavor Liking ^3^	72.0	72.3	71.4	1.23	0.75
Off Flavor ^4^	3.9	4.1	3.9	0.25	0.72
**USDA Select**					
Overall Liking ^3^	68.6 ^b^	72.5 ^a^	70.3 ^ab^	1.23	<0.01
Flavor Liking ^3^	69.7 ^b^	73.2 ^a^	70.8 ^ab^	1.15	<0.01
Toughness ^4^	8.9 ^a^	8.1 ^b^	8.8 ^ab^	0.31	0.02
Off Flavor ^4^	4.5	4.3	4.5	0.23	0.58

^1^ Wet aging days postmortem. ^2^ Largest SEM reported of least squares means. ^3^ 0 = Greatest imaginable disliking; 120 = Greatest imaginable liking. ^4^ 0 = None; 20 = Extremely intense off flavor, extremely tough. ^ab^ Within a row, least squares means without a common superscript differ (*p* < 0.05).

**Table 7:** The interaction of aging and HCW on the sensory attributes of USDA Choice and USDA Select strip loin steaks as rated by consumers.

Attribute	LW 5 ^1^	LW 10 ^1^	LW 14 ^1^	MW 5 ^1^	MW 10 ^1^	MW 14 ^1^	HW 5 ^1^	HW 10 ^1^	HW 14 ^1^	SEM ^2^	*p*-Value
**USDA Choice**											
Overall Liking ^3^	68.1 ^z^	74.6 ^y^	68.8 ^yz^	73.0 ^yz^	74.0 ^yz^	71.9 ^yz^	70.3 ^yz^	70.0 ^yz^	73.1 ^yz^	2.21	0.04
Texture Liking ^3^	65.6 ^b^	75.1 ^a^	68.0 ^ab^	71.1 ^ab^	72.3 ^ab^	70.3 ^ab^	69.4 ^ab^	67.3 ^ab^	70.0 ^ab^	2.52	0.01
Toughness ^4^	10.0 ^a^	7.1 ^b^	8.6 ^ab^	7.3 ^b^	7.5 ^b^	7.8 ^b^	8.7 ^ab^	8.5 ^ab^	8.3 ^ab^	0.59	<0.01
Juiciness ^4^	7.0 ^bc^	8.9 ^a^	6.7^c^	8.6 ^ab^	8.3 ^abc^	7.6 ^abc^	6.8 ^c^	7.5 ^abc^	7.3 ^abc^	0.55	0.01
**USDA** **Select**											
Texture Liking ^3^	68.9 ^yz^	68.9 ^yz^	64.5 ^y^	70.9 ^yz^	70.5 ^yz^	68.1 ^yz^	64.8 ^y^	71.8 ^z^	71.2 ^yz^	2.37	0.01
Juiciness ^4^	7.9 ^ab^	7.9 ^ab^	7.6 ^ab^	8.5 ^a^	7.7 ^ab^	7.9 ^ab^	6.9 ^b^	7.9 ^ab^	8.3 ^ab^	0.49	0.03

^1^ Wet aging days postmortem; LW = Lightweight (296–341 kg), MW = Middleweight (386–432 kg), HW = Heavyweight (466–524 kg). ^2^ Largest SEM reported of least squares means. ^3^ 0 = Greatest imaginable disliking; 120 = Greatest imaginable liking. ^4^ 0 = None; 20 = Extremely tough, extremely juicy. ^abc^ Within a row, least squares means without a common superscript differ (*p* < 0.05). ^yz^ Within a row, least squares means without a common superscript differ (0.05 < *p* < 0.10).

**Table 8:** The effects of hot carcass weight on the sensory attributes of USDA Choice and USDA Select eye of round steaks as rated by consumers.

Attribute	LW ^1^	MW ^1^	HW ^1^	SEM ^2^	*p*-Value
**USDA Choice**					
Overall Liking ^3^	65.6 ^b^	70.2 ^a^	67.4 ^ab^	1.32	<0.01
Flavor Liking ^3^	67.4 ^b^	70.5 ^a^	68.0 ^ab^	1.30	0.04
Texture Liking ^3^	62.9 ^b^	68.8 ^a^	66.2 ^ab^	1.45	<0.001
Juiciness ^4^	6.9 ^b^	8.3 ^a^	7.8 ^a^	0.31	<0.0001
Off Flavor ^4^	4.4	4.1	4.2	0.25	0.51
					
**USDA Select**					
Overall Liking ^3^	64.2	66.5	64.7	1.36	0.21
Flavor Liking ^3^	65.0	66.9	66.7	1.32	0.30
Texture Liking ^3^	63.4	66.2	63.6	1.48	0.10
Off Flavor ^4^	4.7 ^a^	4.2 ^ab^	3.9 ^b^	0.24	<0.01

^1^ LW = Lightweight (296–341 kg), MW = Middleweight (386–432 kg), HW = Heavyweight (466–524 kg). ^2^ Largest SEM reported of least squares means. ^3^ 0 = Greatest imaginable disliking; 120 = Greatest imaginable liking. ^4^ 0 = None; 20 = Extremely intense off flavor, extremely juicy. ^ab^ Within a row, least squares means without a common superscript differ (*p* < 0.05).

**Table 9:** The effects of aging on the sensory attributes of USDA Choice and USDA Select eye of round steaks as rated by consumers.

Attribute	5 ^1^	10 ^1^	14 ^1^	SEM ^2^	*p*-Value
**USDA Choice**					
Overall Liking ^3^	67.7	68.8	66.8	1.32	0.35
Flavor Liking ^3^	68.4	69.8	67.6	1.30	0.23
Texture Liking ^3^	66.0	66.9	65.1	1.45	0.49
Juiciness ^4^	7.5	7.7	7.8	0.31	0.49
Off Flavor ^4^	4.3	4.2	4.2	0.25	0.83
**USDA Select**					
Overall Liking ^3^	66.0 ^ab^	62.9 ^b^	66.5 ^a^	1.36	0.02
Flavor Liking ^3^	66.7 ^ab^	64.2 ^b^	67.6 ^a^	1.32	0.02
Texture Liking ^3^	65.4 ^ab^	62.0 ^b^	65.7 ^a^	1.48	0.02
Off Flavor ^4^	4.3	4.3	4.2	0.24	0.70

^1^ Wet aging days postmortem. ^2^ Largest SEM reported of least squares means. ^3^ 0 = Greatest imaginable disliking; 120 = Greatest imaginable liking. ^4^ 0 = None; 20 = Extremely intense off flavor, extremely juicy. ^ab^ Within a row, least squares means without a common superscript differ (*p* < 0.05).

**Table 10:** The interaction of aging and HCW on the sensory attributes of USDA Choice and USDA Select eye of round steaks as rated by consumers.

Attribute	LW 5 ^1^	LW 10 ^1^	LW 14 ^1^	MW 5 ^1^	MW 10 ^1^	MW 14 ^1^	HW 5 ^1^	HW 10 ^1^	HW 14 ^1^	SEM ^2^	*p*-Value
**USDA Choice**											
Toughness ^3^	11.1^a^	10.6 ^ab^	10.2 ^abc^	8.4 ^c^	9.0 ^bc^	9.2 ^bc^	8.8 ^c^	8.5^c^	9.9 ^abc^	0.58	<0.05
**USDA** **Select**											
Toughness ^3^	10.8 ^ab^	10.7 ^abc^	10.5 ^abc^	9.9 ^abc^	10.3 ^abc^	9.0 ^c^	9.2 ^bc^	11.4 ^a^	10.5 ^abc^	0.58	<0.01
Juiciness ^3^	7.0 ^bc^	6.2 ^bcd^	7.2 ^abc^	6.8 ^bc^	6.0 ^cd^	8.9 ^a^	7.0 ^bc^	4.9 ^d^	7.8 ^ab^	0.54	0.02

^1^ Wet aging days postmortem; LW = Lightweight (296–341 kg), MW = Middleweight (386–432 kg), HW = Heavyweight (466–524 kg). ^2^ Largest SEM reported of least squares means. ^3^ 0 = None; 20 = Extremely tough, extremely juicy. ^abcd^ Within a row, least squares means without a common superscript differ (*p* < 0.05).

## Data Availability

The original contributions presented in the study are included in the article, further inquiries can be directed to the corresponding author.
